# Supplementary material for: Effects of Acute and Chronic Resistance Exercise on the Skeletal Muscle Metabolome
Source: Metabolites. 2022 May 16;12(5):445. doi: 10.3390/metabo12050445 (PMC9142957; doi:10.3390/metabo12050445)

Supplementary Figure S1: Metabolite levels for all 6 participants at the three sampling points shown for all metabolites with significant changes listed in Table 1.

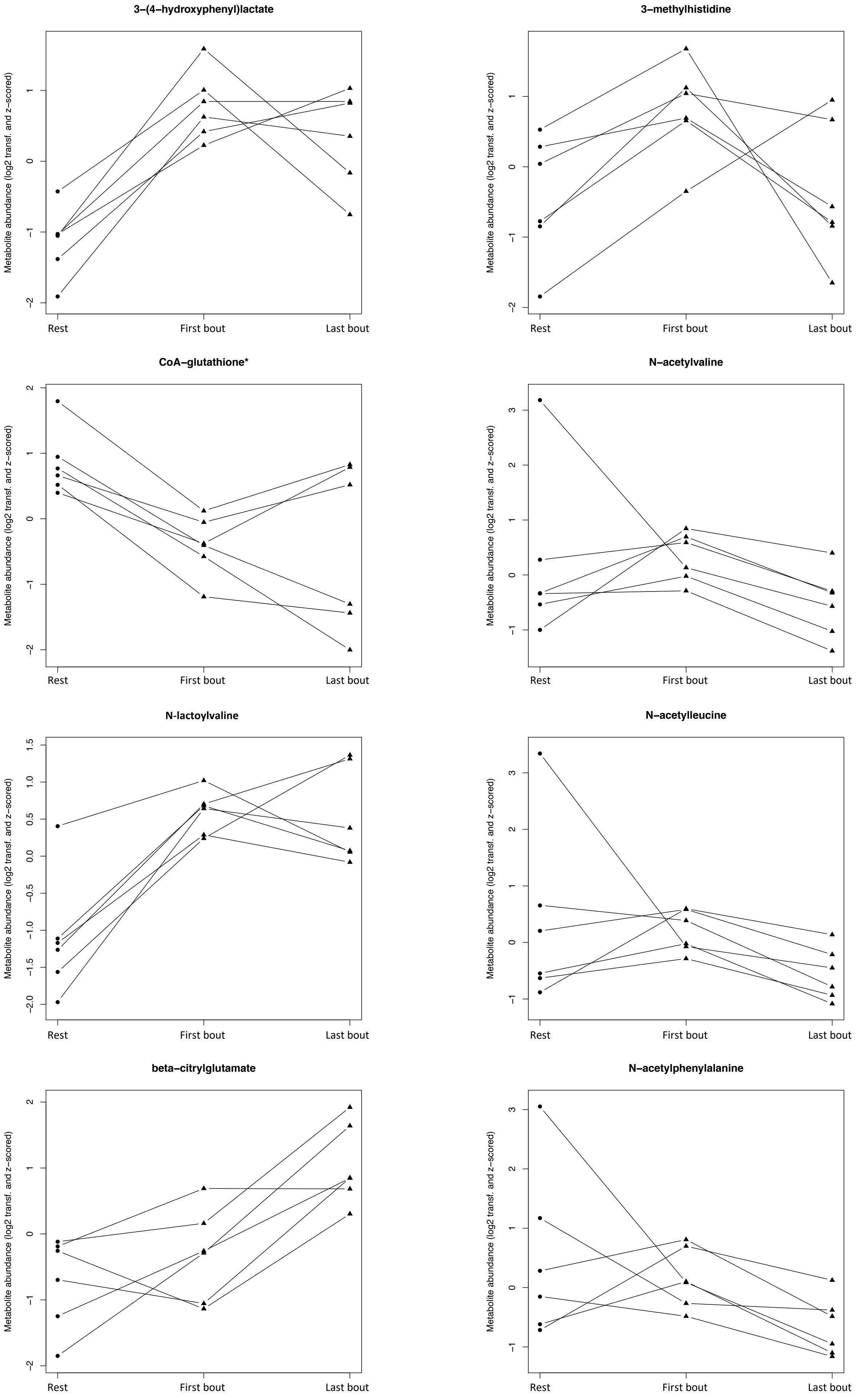

Supplementary Figure S1: continued.

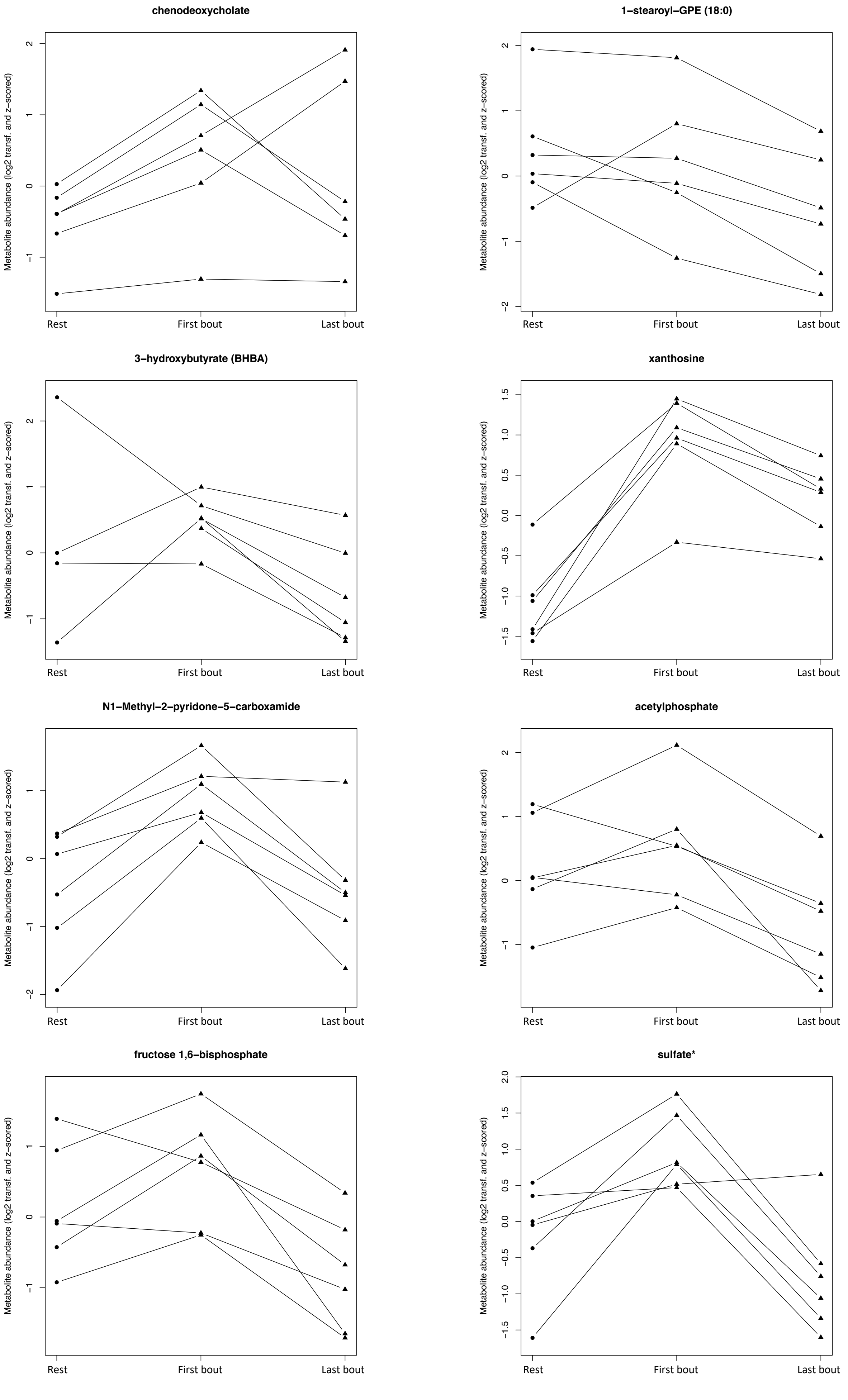

Supplement: Supplementary file 1 [file metabolites-12-00445-s001.zip › SupplementaryFigureS1_v2.pdf]
